# Supplementary material for: The interactome of intact mitochondria by cross-linking mass spectrometry provides evidence for coexisting respiratory supercomplexes
Source: Mol Cell Proteomics. 2017 Dec 8;17(2):216–32. doi: 10.1074/mcp.RA117.000470 (PMC5795388; doi:10.1074/mcp.RA117.000470)
Supplement: Supplemental Data [file supp_RA117.000470_133922_0_supp_23978_fzffwf.zip › spectra_annotation/mito_spectra_annotation/396-3-5-1-4-1.pdf]

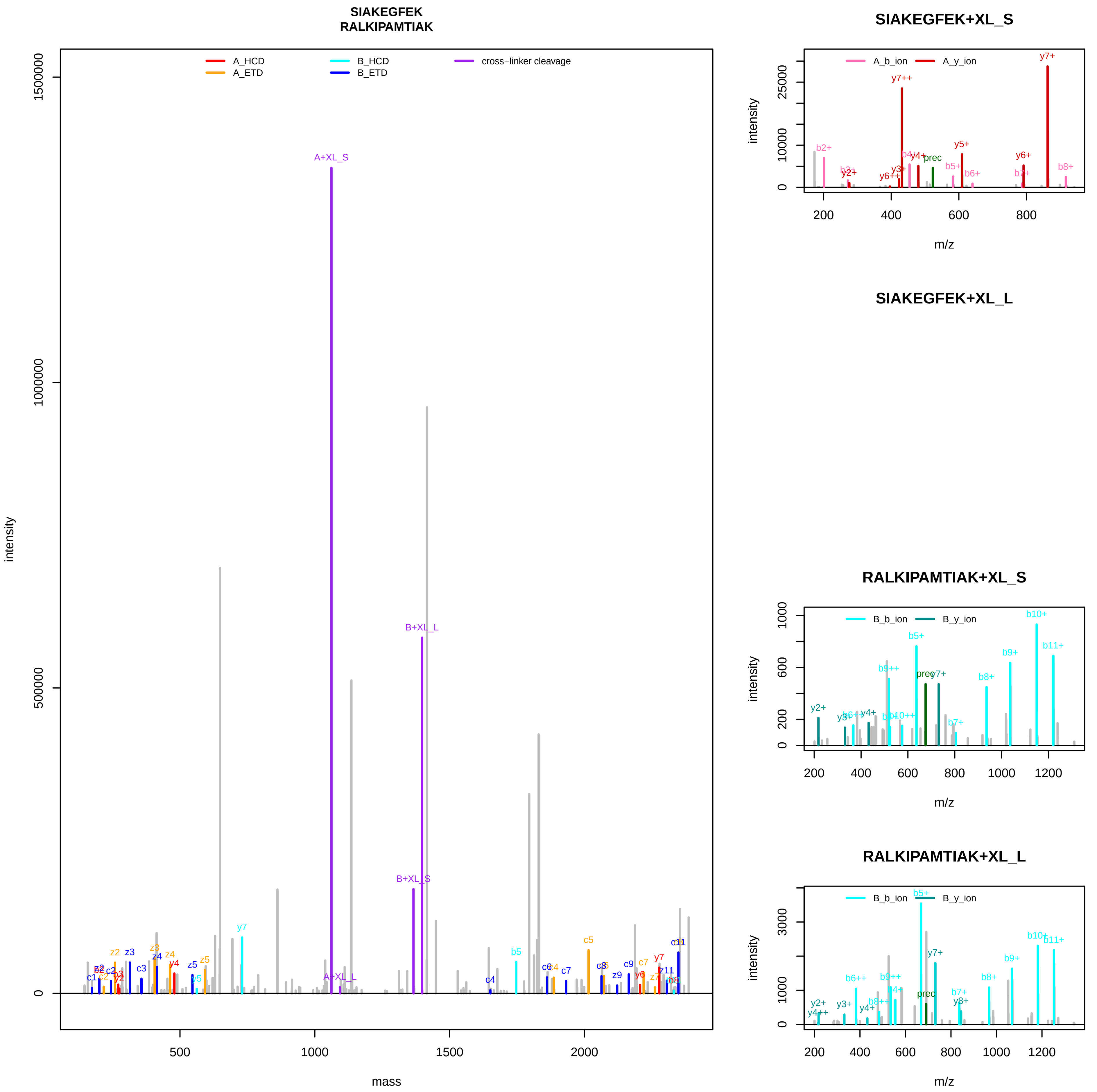

SIAKEGFEK
RALKIPAMTIAK
A_HCD
A_ETD
B_HCD
B_ETD
cross−linker cleavage
A+XL_S
B+XL_L
B+XL_S
A+XL_L
intensity
mass
0
500000
1000000
1500000
500
1000
1500
2000
SIAKEGFEK+XL_S
A_b_ion
A_y_ion
y7+
y7++
y5+
y6+
prec
b2+
b5+
b6+
b8+
m/z
SIAKEGFEK+XL_L
RALKIPAMTIAK+XL_S
B_b_ion
B_y_ion
b10+
b5+
b9+
b11+
b9++
b8+
y2+
y3+
y4+
b7+
RALKIPAMTIAK+XL_L
b6++
b10+
b11+
y7+
prec
b8+
